# Music-based intervention drives paretic limb acceleration into intentional movement frequencies in chronic stroke rehabilitation

**DOI:** 10.3389/fresc.2022.989810

**Published:** 2022-10-03

**Authors:** Tristan Loria, John de Grosbois, Catherine Haire, Veronica Vuong, Nina Schaffert, Luc Tremblay, Michael H. Thaut

**Affiliations:** ^1^ Music and Health Research Collaboratory (MaHRC), Faculty of Music, University of Toronto, Toronto, ON, Canada; ^2^ Baycrest Health Sciences, Rotman Research Institute, Toronto, ON, Canada; ^3^ Department of Movement and Training Science, Institute for Human Movement Science, University of Hamburg, Hamburg, Germany; ^4^ BeSB GmbH Berlin Sound Engineering, Berlin, Germany; ^5^ Faculty of Kinesiology and Physical Education, University of Toronto, Toronto, ON, Canada; ^6^ Faculty of Medicine, University of Toronto, Toronto, ON, Canada

**Keywords:** stroke, sonification, limb acceleration, rehabilitation, motor control

## Abstract

This study presented a novel kinematic assessment of paretic limb function “online” during the actual therapeutic exercisers rooted within the acceleration domain. Twenty-eight patients at chronic stroke stages participated in an auditory-motor intervention mapping reaching movements of the paretic arm unto surfaces of large digital musical instruments and sound tablets that provided rhythmic entrainment cues and augmented auditory feedback. Patients also wore a tri-axial accelerometer on the paretic limb during the nine-session intervention. The resulting acceleration profiles were extracted and quantified within the frequency domain. Measures of peak power and peak width were leveraged to estimate volitional control and temporal consistency of paretic limb movements, respectively. Clinical assessments included the Wolf Motor Function Test and Fugl-Meyer – Upper Extremity subtest. The results showed that peak power increased significantly from Session 1 to Session 9 within oscillatory frequency ranges associated with intentional movement execution (i.e., 4.5 Hz). Decreases in peak width over time provided additional evidence for improved paretic arm control from a temporal perspective. In addition, Peak width values obtained in Session 1 was significantly correlated with pre-test Fugl-Meyer – Upper Extremity scores. These results highlighted improvements in paretic limb acceleration as an underlying mechanism in stroke motor recovery and shed further light on the utility of accelerometry-based measures of paretic limb control in stroke rehabilitation.

The data reported here was obtained from a larger clinical trial: https://clinicaltrials.gov/ct2/show/NCT03246217 ClinicalTrials.gov Identifier: NCT03246217.

## Introduction

Stroke adversely affects individuals, their families, and society at large. Motor impairments including upper-limb dysfunction are among the most debilitating and persistent post-stroke symptoms (1). Goal-driven movements including reaching can become slow and indirect (2), which strongly predicts whether patients resume pre-stroke activities of daily living (ADL) (3, 4). If a functional threshold of recovery is not reached, patients often resort to compensatory movements (5) or simply refrain from using the affected limb (6). Such adaptations subsequently diminish well-being and limit social participation (7). Critically, regaining movement capacity is particularly challenging in the chronic phase of recovery (8–10). Although some spontaneous recovery can occur (11), such recovery is use-dependent (12). Therefore, the focus of physical rehabilitation is restoring functionality in the affected limbs and sustaining sensorimotor improvements over time (13).

Auditory-motor based interventions (AMIs) are known to benefit paretic limb function in chronic stroke. Previous studies have reported improved paretic limb function by mapping reaching movements unto large surface musical instruments (e.g., percussion, keyboard, etc.) that provided augmented auditory feedback at movement endpoints and predictable “feedforward” timing cues when embedded in rhythmic auditory stimulation (14–16). More recently, Haire et al. (17) found functional pre- vs. post-test paretic limb gains on the Fugl-Myer Upper-Extremity (FM-UE) and Wolf Motor Function tests (WMFT) following training using an AMI technique called Therapeutic Instrumental Music Playing (TIMP). In the Haire et al. study, typical acoustic instruments (e.g., drums, piano) and rhythmic auditory stimulation was combined with a music technology device consisting of a digital touch-responsive aiming surface that provided movement-related auditory feedback (i.e., sonification) as anticipatory timing cues during paretic limb movements. Patients in the Haire et al. study also wore accelerometers on the paretic limb to further quantify paretic limb function.

Accelerometers can provide additional mechanistic knowledge regarding motor recovery in stroke rehabilitation. To this end, specific accelerometry-based outcome measures have been developed to quantify paretic limb use. These measures have focused on quantifying movement magnitudes (i.e., limb speed as a function of distance, see ref. 18) and variability (i.e., fluctuations in movement speed, see ref. 19) to further understand paretic limb function. Additional measures have concentrated on quantifying the amount of paretic limb involvement in bilateral tasks such as writing and cutting (20). These metrics are often measured and assessed while the patient behaved in typical ADLs outside of the laboratory to understand real-world paretic limb use and recovery (21, 22). However, such an approach does not offer clues about specific motor control changes in movement execution occurring “online” during the actual rehabilitation process. Further quantifying accelerometry-based kinematics could offer clinicians objective data for selecting appropriate training regimes, and further understanding the effectiveness of AMIs.

**Figure 1:**
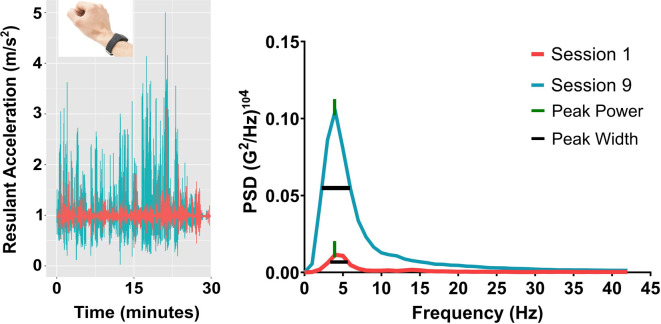
Summary of the acceleration analysis. On the left side are the raw acceleration profiles for **Session 1** (magenta) and 9 (teal) along the resultant movement axis. On the right side is the resulting power spectral density function showing peak power and peak width for the same participant.

Our research team has recently developed a frequency-based assessment of limb acceleration to objectively quantify upper-limb sensorimotor control in healthy adults. Based on oscillation frequency decomposition methods (23), it is possible to extract critical sensorimotor processes from acceleration traces that reflects purposeful feedback-driven movement (see Figure 1) that has not been the focus of previous accelerometry-based metrics (18–22). Specifically, the relative contributions of different oscillation frequencies (i.e., certain timescales) from an acceleration signal can be extracted *via* what is known as a normalized power spectral density function (24–26). This kinematic-based approach has implications for assessing functional motor recovery, given that different oscillations frequencies distinguish between intentional (i.e., movements around 4 Hz) and unintentional ancillary movements (e.g., movement jitter, micro-tremor, “overflow” movements etc.). The two measures that quantify the intentionality of upper-limb movements are referred to as peak power and peak width.

Peak power and peak width may be useful for quantifying motor recovery in stroke rehabilitation, given previous reports demonstrating the utility of accelerometry-based metrics. Increases in peak power localized to peaks in the acceleration trace represent greater average acceleration at specific rates. More specifically, peak power measures the rate of change in arm movement speed by identifying the greatest limb acceleration value occurring during the entire training session. In non-clinical studies, peak power magnitude was greater when visual feedback of the reaching environment was available compared to when visual feedback was unavailable, with the greatest peak in the power spectrum localized around 4 Hz. Given that peak power occurring around 4 Hz was found when comparing between vision and no-vision aiming conditions, fluctuations in peak power magnitude may reflect intentional sensorimotor control of goal-driven movement (24, 25). Relatedly, peak width refers to the bandwidth of variability across the peak power spectrum. Increases in peak width indicate greater variability in the acceleration profile of arm movements, whereas reductions in peak width represent more consistent acceleration rates (24, 25). However, peak power and peak width have yet to be applied within motor re-learning settings such as stroke rehabilitation. Within rehabilitation contexts, changes in both measures occurring within movement frequencies around 4 Hz would indicate the presence of feedback-driven movements directly related to motor function. As such, peak power and peak width may be useful metrics for assessing patient progress during training and global effects of an intervention.

The rhythmic aspects of AMI approaches (i.e., rhythmic auditory stimulation, sonification, etc.) (13–17) provide an ideal laboratory for studying movement acceleration parameters using kinematic assessments. Indeed, AMIs such as TIMP may facilitate temporal control (e.g., acceleration) of paretic limb movements by mapping paretic arm trajectories to the touch surfaces of acoustical and digital instruments/devices which create auditory feedback upon contact (15, 17, 27, 28). Therefore, the present study utilized frequency-based analyses as dynamic movement assessments, while clinical pre- and post-test assessments were obtained *via* the FM-UE and WMFT in the same cohort. In the early phases of the AMI, it was hypothesized that peak width values would initially be above the volitional control frequencies in tandem with low peak power. As training progressed, it was expected that peak width values would narrow to volitional ranges (i.e., 4 Hz), with peak power magnitude increasing. Based on previous accelerometry-based studies (18–21), it was predicted that improvements in the dynamic acceleration-based measures may show moderate to strong relationships between FM-UE and WMFT scores at pre- and post-test timepoints (20). Evidence in favour of these hypotheses may reveal acceleration-based mechanisms underlying motor recovery during the actual execution of therapeutic exercises, which test-based clinical assessments do not measure.

## Materials and methods

### Participants

This study was part of a larger register clinical trial (see: ref. 20 and Clinical Trials.gov ID#NCT03246217). The parent study split participants into three groups who received identical TIMP training for the first thirty minutes of the intervention (i.e., the data reported here). In the remaining fifteen minutes of the parent study, participants engaged in different forms of mental practice (17, 27). The initial sample size of the parent study was derived using power analyses. Specifically, the FM-UE has a standardized response mean of 1.42 (95% CI = 1.19, 1.80) whereas the standardized response mean for the WMFT is 1.3 (95% CI 1.03, 1.67) (see ref. 29). At a *p* value of .05 and 80% power, it was determined that 24 participants would be required to detect a standardized difference for the FM-UE and WMFT in the parent study. Due to technical issues related to the wrist-worn accelerometer (i.e., accelerometer off during training), data from twenty-eight (i.e., 14 females, age range 33–76, mean age 55.9, *SD* = 17) of the thirty community-dwelling adults who completed the parent study were analyzed and reported here.

All participants had sustained a unilateral stroke an average of 66.9 months (*SD* = 15.2) prior to participating. Participants were primarily recruited from the multicultural Greater Toronto Area. Inclusion criteria were hemiparesis, with at least minimal volitional movement of the affected limb, adequate language comprehension, and typical neurocognitive function. Exclusion criteria was enrolment in an alternate upper extremity rehabilitation program, comorbid neurological disorder, or injections for spasticity less than three months before training. The study was approved by the University of Toronto Research Ethics Board (Protocol number 33418). All participants provided written informed consent to the investigator, prior to participation, in accordance with the Declaration of Helsinki. Demographic data has been provided in Table 1.

**Table 1:** Demographic information for the participants. Mean values are shown with standard deviation values provided in brackets. Note MoCA refers to Montreal Cognitive Assessment.

Measure	Mean (SD)
Age in Years	55.9 (12.3)
Years of Education	16.3 (7.7)
MoCA Score	26.8 (3.2)
Months since Stroke	66.9 (60.7)
# Hemorrhagic stroke	10
# Ischemic stroke	18
# Lesion on right side	17
# Lesion on left side	11
# Lesion in frontal lobe	7
# Lesion in brainstem	6
# Middle cerebral artery territory	10
# Subcortical	5

### Procedure

All participants completed nine sessions over three weeks (i.e., three sessions per week). Exercises were designed to facilitate retraining of functional movement patterns involving proximal and distal control. The main digital auditory devices used for mapping rhythmically cued movements were a Yamaha CP40 Stage Piano and DTX Drums (Yamaha Corporation, Hamamatsu, Shizuoka, Japan). A specially designed digital interactive sound tablet, known as the Sonification Arm Training Apparatus (SONATA), provided real-time auditory feedback of limb tracing, and pointing movements (17, 27, 28). A digital metronome device was used to create anticipatory timing cues for motor entrainment (30, 31) and ascertain the participant's preferred movement frequency. This approach provided the participants with a consistent rhythmic template for gauging movement trajectories in accordance with spatiotemporal constraints.

Participants performed rhythmically-cued movements during training. Instruments and devices were positioned spatially as movement end points to target specific movement types including: 1) shoulder abduction/adduction, 2) elbow flexion/extension, 3) wrist pronation/supination, 4) selective finger dexterity, and 5) upper trunk rotation. Each session was subdivided into two blocks of fifteen minutes in which three minutes were dedicated to each of the five exercise foci described above. Repetition rate varied slightly across participants, depending on ability. To gather the kinematics of the upper limb in the acceleration domain, participants wore a GENEActiv Action accelerometer (Activinsights, Cambridgeshire, UK) on the paretic limb throughout each training session. The accelerometer sampled at 1,000 Hz with a range of ±16 g. Clinical assessment data (i.e., FM-UE and WMFT) were obtained on the day prior to the first training session (i.e., pre-test) and the day after the last training session (i.e., post-test). For the FM-UE, participants completed the A-D motor function assessments (i.e., upper extremity, wrist, hand, and coordination/speed) for a total potential score of 66.

### Data analysis

#### Data reduction

Data reduction was completed using the Anaconda Distribution of the Python 3 programming language using functions available within the SciPy library (32). Accelerometer data was obtained for the three orthogonal axes (i.e., x, y, and z). Because upper-limb function varied across individuals, resultant acceleration was chosen as the primary substrate for the frequency-domain analyses. These resultants were computed as the square-root of the sum of the squared values of each axis and time-point. To reduce noise introduced by this process, the time-series were low-pass filtered using a second-order, dual-pass Butterworth filter, using a cut-off frequency of 40 Hz. Power-spectral density estimates (PSDs) were computed from these filtered resultant time-series using the pwelch function (see Figure 1). The pwelch function was applied using the following input parameters: 1 s sliding intervals, a 50% interval overlap, a Hanning truncation window, and linear detrending for each individual. This procedure returned a median estimate of PSD across all processed intervals. The median was chosen to minimize artifacts having an influence on the frequency domain estimates. Two main outcomes were quantified from each timeseries' PSD: 1) the magnitude of the largest peak in the spectrum (i.e., peak power); and 2) the width in Hz of the largest peak at 50% of its height (i.e., peak width). The properties of the largest peak were quantified using the findpeaks function. The peak power values were reported in units of 1/1000 (i.e., (G^2^/Hz)^104^). Peak power and peak width were extracted per session and per participant and averaged to compare across sessions (e.g., Session 1 vs. Session 9).

### Data processing

Due to mechanical, software, and/or human error, data was not gathered for 11 sessions across the 259 total sessions in the experimental design (i.e., 4% of the data was missing). Data from an additional 16 sessions were further excluded upon outlier analyses (i.e., peak power values >2 SD from the mean). The average values for the individual sessions were used as replacement data for that session and participant in cases of missing or outlier data.

### Statistical testing

#### Frequency-based analyses

Normality was first assessed using histograms. Following the confirmation of normality, the effect of the intervention on peak power and peak width were examined *via* repeated measures analysis of variance (ANOVA). Separate ANOVAs for each variable were conducted with session (i.e., 1–9) entered as the within-subjects factor. When sphericity had been violated, Greenhouse-Geisser corrections were applied to the degrees of freedom and reported to one decimal place. Given the continuous nature of the effect of session, a polynomial trend analysis was used to quantify changes in peak power and peak width across sessions (e.g., linear, quadratic, cubic, quartic, etc.). In the presence of significant polynomial trends, pointed paired samples *t-tests* were used to highlight associated between session differences.

#### Clinical assessment analyses

Complete pre- and post-test clinical assessments using FM-UE and WMFT were reported elsewhere (17, 27). Initial data screening and processing determined both FM-UE and WMFT data did not meet parametric analyses assumptions. For both measures, differences between the pre- and post-test scores were assessed using the Wilcoxon signed-ranked test.

### Relationships between measures

Pearson correlation analyses compared baseline (i.e., pre-test) and post-test FM-UE scores to peak power and peak width values obtained during Session 1 and 9, respectively. Correlations also compared WMFT baseline (i.e., pre-test) and post-test scores to peak power and peak width values obtained during Sessions 1 and 9. Because two comparisons were conducted using the same data (i.e., Peak Power Session 1 vs. FM-UE pre-test; Peak Power Session 1 vs. WMFT pre-test), Bonferroni corrections were applied to the alpha value which yielded an adjusted significance threshold of *p *=* *0.03. To further explore the link between changes in peak power, peak width, FM-UE, and WMFT, peak power and peak width values measured in Session 1 were subtracted from Session 9 to yield *Δ* peak power and *Δ* peak width. Similarly, pre-test FM-UE and WMFT-FA scores were subtracted from the post-test scores to obtain *Δ* FM-UE and *Δ* WMFT scores. These *Δ* scores were subsequently assessed qualitatively using scatter-plots to understand potential mechanisms linking changes in paretic limb acceleration and clinical assessment scores. All statistical contrasts mentioned above were performed using SPSS version 25 (SPSS Statistics, IBM, Armonk, New York, USA).

## Results

### Accelerometry

**Figure 2:**
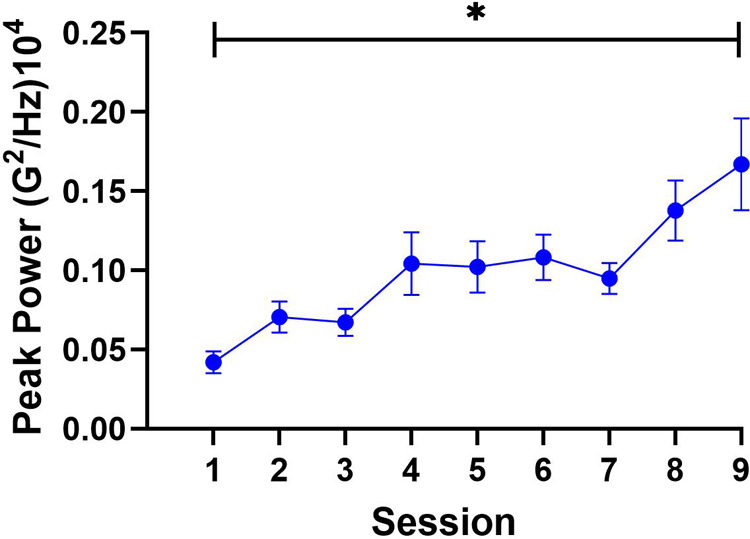
The findings for peak power. Peak power was greater in **Session 9** compared to **Session 1**, which supports the effectiveness of the intervention. Note: Error bars denote standard error of the mean, * = significance at <0.05.

The results of the repeated-measures *ANOVA* for peak power revealed a main effect of session, *F* (3.5, 95.4) = 8.8, *p* < 0.001, $n_{p}^{2}$ = 0.25. Follow-up trend analysis revealed significant linear, *F* (1, 216) = 60.2, *p* <*** ***0.001, $n_{p}^{2}$ =*** ***0.22, and cubic *F* (1, 216), = 4.9, *p* = 0.03, $n_{p}^{2}$ = 0.02 trends. The relatively large linear effect suggested that patients demonstrated consistent improvement in paretic limb control during training. Given the strength of the linear trend and hypotheses outlined above, the linear trend was further examined using a *t-test* to contrast differences in peak power between Sessions 1 and 9 (see Figure 2). The results showed that movements of the paretic limb increased in peak power, *t* (27) = 4.8, *p* = 0.001, 95% CI [−0.2, - 0.7] from Session 1 (M = 0.04 G^2^/Hz, SD = 0.03) to Session 9 (M = 0.2 G^2^/Hz, SD = 0.2), with a magnitude of peak power increase of 0.16 G^2^/Hz. This finding suggests that paretic limb movements were executed with greater peak acceleration at frequencies associated with intentional voluntary control following the intervention.

**Figure 3:**
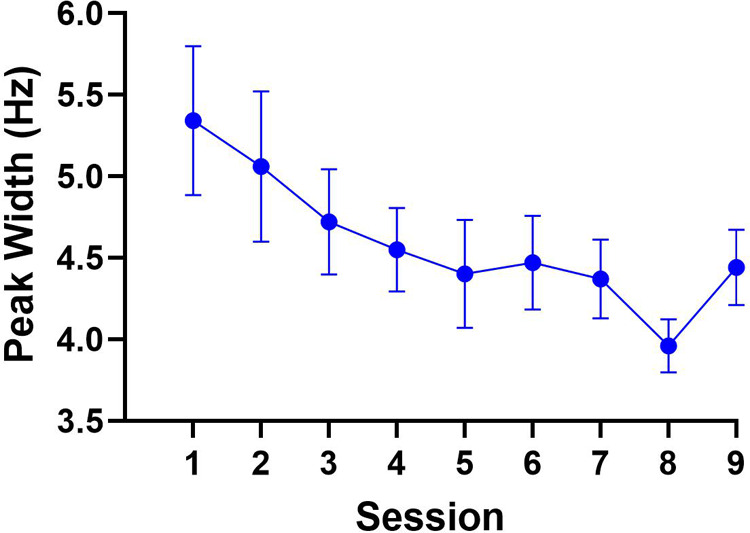
The results for peak width. Although not significant, peak width numerically decreased from **Sessions 1 to 9**, localized to frequency ranges commonly associated with voluntary motor control.

The repeated-measures ANOVA computed for peak width yielded a main effect of session, *F* (9.5, 104.8) = 2.6, *p* < 0.05, $n_{p}^{2}$ = 0.09. The polynomial trend analysis revealed the linear model as the only significant trend, *F* (1, 216) = 15.9, *p* = 0.001, $n_{p}^{2}$ = 0.07, indicating that paretic limb movements were executed with reduced temporal variability over the course of the intervention. Post-hoc tests for the linear trend contrasted peak width between Sessions 1 and 9. This analysis, however, did not reveal a significant difference, *t* (27) = 2.0, *p *= 0.08, 95% CI [-0.01, 1.8], between the width of the peak at Session 1 (M = 5.3 Hz, SD = 2.4) compared to Session 9 (M = 4.4 Hz, SD = 1.2). Yet, the presence of the significant linear trend, which may have been influenced by the peak width in Sessions 7 and 8 (i.e., 4.37 and 3.96, respectively), indicated that improvements in temporal consistency of paretic limb movements may be anticipated with additional training sessions. The results are illustrated in Figure 3.

### Clinical assessments

The clinical outcomes further revealed a beneficial impact of the intervention. Detailed results are reported elsewhere (17, 27). FM-UE analysis revealed a statistically significant median score improvement between pre- (i.e., 31.5) and post-test (i.e., 38.5), *z* = 2.53, *p* = 0.01, *r* = 0.61. WMFT scores also significantly improved between pre- (i.e., 28.0) to post-tests (i.e., 32.5), *z* = 2.75, *p* = 0.01, *r* = 0.62.

### Relationships between clinical and kinematic measures

Correlations analyses compared pre- and post-test FM-UE and WMFT to Session 1 and Session 9 peak power and peak width values, respectively. The results for the correlation analyses are shown in Table 2. This analysis revealed a significant negative correlation between FM-UE pre-test and peak width S1, *r* (26) = −0.43, *p* = 0.02.

**Table 2:** Correlational analyses between clinical and accelerometry-based metrics. Values marked with * indicate a statistically significant difference at *p* < 0.03.

	Peak Power S1	Peak Power S9	Peak Width S1	Peak Width S9
FM-UE Pre-test	0.16	−	−0.43*	−
FM-UE Post-test	−	0.05	−	−0.11
WMFT Pre-test	−0.04	−	−0.31	−
WMFT Post-test	−	−0.05	−	−0.18

Note: S, Session.

**Figure 4:**
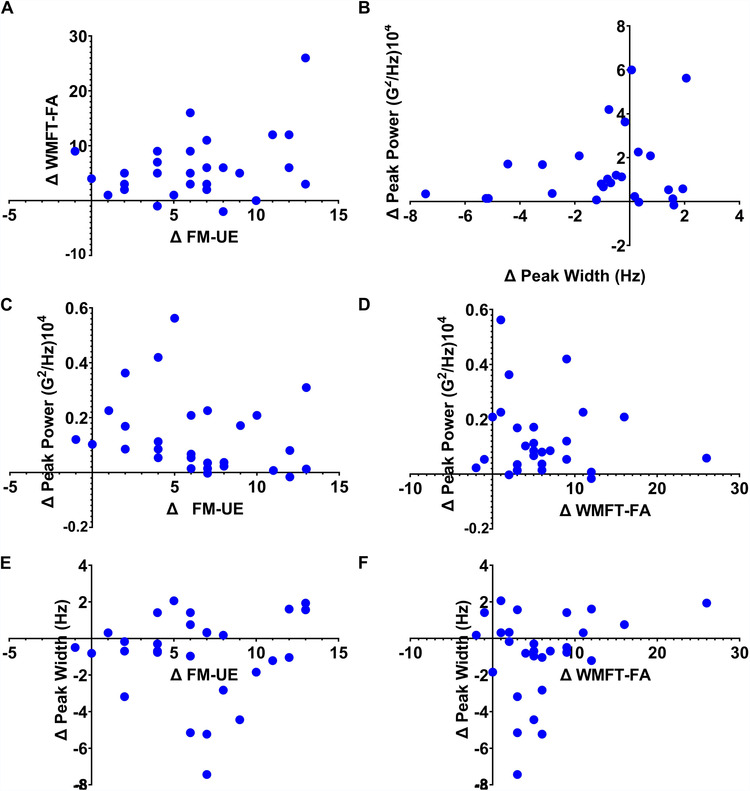
The results for relationship between measures. The relationship between WMFT and FM-UE scores (i.e., Panel **A**) as well as between *Δ* peak power and *Δ* peak width (i.e., Panel **B**) are shown. *Δ* peak power and *Δ* FM-UE and *Δ* WMFT are shown in panels **C and D**, respectively. Lastly, Panels **E and F** show the relationship between *Δ* peak width and *Δ* FM-UE and *Δ* WMFT.

The relationship between clinical and accelerometry-based metrics are further shown in Figure 4. First, it is important to note that patients with the largest *Δ* peak width had relatively small *Δ* peak power. Further inspection of the relationships between *Δ* peak power, *Δ* FM-UE, and *Δ* WMFT revealed that improvements in both clinical measures could be localized to the lower range of peak power improvements (i.e., peak power values around. 2 G^2^/Hz^104^; see Figure 3). Regarding *Δ* peak width, participants who showed intermediate improvements in FM-UE scores (i.e., *Δ* between 5 and 10) showed the greatest numerical improvements in peak width wherein peak width narrowed towards the volitional control range following the intervention. Like *Δ* peak power, the relationship between *Δ* peak width and *Δ* WMFT scores showed a tight clustering of patients who demonstrated improved peak width with smaller *Δ* WMFT improvement scores. Thus, these patterns potentially highlighting the suitability of frequency-based accelerometry assessments for assessing underlying motor control mechanisms that may have contributed to improved clinical assessment scores following rehabilitation.

## Discussion

This study applied a frequency-based kinematic assessment of paretic limb control within TIMP as the chosen AMI. Twenty-eight patients completed nine training sessions involving the provision of anticipatory rhythmic auditory cues and sonification feedback while executing upper-limb movements on digital sound devices and acoustic instruments. The results showed increases in peak power between Sessions 1 and 9, indicating that paretic limb movements were executed with greater acceleration during the actual intervention exercises. Increases in peak power also coincided with overall reductions in peak width across sessions demonstrating stronger, yet more consistent, acceleration profiles that trended towards oscillatory movement frequencies associated with intentional goal-directed movement. Furthermore, the changes in the frequency-based assessments during movement execution were reflective of changes in clinical assessment scores between pre- and post-tests, with moderate correlations revealed for Session 1 peak width and FM-UE pre-test scores. The relevance of these findings for mechanisms of motor recovery and future clinical assessments are discussed below.

Frequency-based assessments reported here may be useful to the field of stroke rehabilitation. Indeed, previous work has demonstrated a clinical link between limb acceleration and motor dysfunction. For example, disruptions within paretic limb acceleration can reduce the smoothness of reaching movements (33). Compared to the unaffected limb, paretic limb movements were executed with lower average and peak acceleration (34), which may reduce the frequency of paretic limb use (35). Thus, reduced limb acceleration regulation may represent a mechanism underlying motor dysfunction in stroke. Critically, the present study showed a relationship between peak width in Session 1 and FM-UE pre-test scores which may be interpreted as evidence that variability in paretic limb acceleration relates to the degree of motor impairment. However, given the strength of the correlation, such an interpretation must be interpreted with caution. Nevertheless, effective interventions may elect to emphasize exercise structures that drive modulations in the speed of functional movements during therapeutic exercises. AMIs represent one potential candidate for such therapeutic exercises. The use of TIMP in the present study may have implicitly driven the paretic arm into greater and more consistent accelerations due to its cyclical and repetitive structure embedded into anticipatory entrainment cues (i.e., rhythmic beats), which is supported by results of previous AMIs (14–17, 27, 28). To test this speculative hypothesis, future therapeutic exercises may also employ frequency-based assessments during the actual execution of training exercises to measure motor recovery within and outside of AMIs.

Measures of peak power and peak width represent a means to directly measure the intentionality of motor execution. Significant increases in peak power highlight that paretic limb movements were executed with greater acceleration during the intervention, suggesting that functional improvements in paretic limb control had occurred. Increases in peak power were further supported by previous accelerometry-based studies showing that the magnitude of paretic limb movements improved during ADLs outside of the laboratory (18, 21, 22). By quantifying the greatest peak in the acceleration of paretic limb movements peak power is a representation of movement intensity. That is, peak power measures dynamic motor behaviour in a way that static clinical assessments do not. The FM-UE assessment in particular measures the ability of the paretic limb to engage in functional movements in general but does not directly quantify the speed or intensity of such movements (36, 37). The specific contribution of peak power as an assessment tool relates to quantifying the intensity of paretic limb movements, and how such intensity may be altered during and following therapy. Thus, peak power may provide a measure of motor control progress in patient recovery “online” during therapy, which other standard clinical assessments cannot capture.

Additional functional changes may also have been detectable though peak width narrowing across the intervention, localized within frequency ranges commonly associated with goal-directed behaviour (38–40). Regarding the relationship between peak power and peak width, the results showed that reductions in peak width tended to be associated with large increases in peak power, with peak power values from Session 1 showing moderate correlations with participant's pre-test FM-UE scores. Indeed, reductions in paretic limb acceleration variability were expected given the close association between paretic limb function and acceleration variability reported in the literature (19, 20). It may therefore be hypothesized that as paretic limb motions stabilized from a temporal perspective (i.e., peak width narrowing over time), patients may have began executing movements with greater intensity (i.e., increased peak power). Therefore, the present study may highlight the suitability of using frequency-based accelerometry assessments in tandem with previously developed acceleration metrics (22) to assess paretic limb recovery in chronic stroke. Although comparing peak power and peak width to previously developed accelerometry metrics is beyond the scope of the present study, future investigations may elect to compare peak power and peak width to acceleration metrics reported by Lang and colleagues (18–22) in the same patient cohort to further elucidate the best approaches for quantifying kinematics of motor recovery in the acceleration domain.

Prior to concluding, it is worth considering the major limitations of the present study. As shown in Table 2, the strongest correlation between the accelerometry and clinical assessments is moderate with the remaining correlations displaying weak to no relationships. As such, the correlational analyses should be interpreted with caution until further validation is provided. The weak to moderate correlations may have been observed because peak power and peak width were developed to measure feedback-driven motor behaviour in healthy adults (24, 25). During TIMP training, patients **likely** engaged in ballistic forms of paretic limb control rather than controlling the limb online (41, 42), which would be expected to reduce the strength of peak power and peak width measures. Alternatively, increases in the overall peak acceleration of the limb (i.e., movement intensity) may not reflect paretic limb function as closely as acceleration magnitude metrics developed by Lang and colleagues (i.e., moving faster over a greater distance, see ref. 18–22). Peak power and peak width also currently require technological literacy to compute both variables, which may prove challenging for therapists and clinicians. Finally, patients were not for screened musculoskeletal disorders of the upper-limbs, which may have impacted the degree of motor function and thus the accelerometry-based assessments.

In summary, accelerometry-based assessments (i.e., peak power and peak width) delivered complementary dynamic profiles on movement kinematics to document progress during the actual execution of therapeutic exercises in a way that commonly used pre- and post-test assessments cannot (e.g., FM-UE, WMFT, etc.). Regarding feasibility, accelerometry-based assessments can be implemented within clinical settings with relatively small investments in technical equipment and therefore are accessible for patients and therapists. Lastly, this study showed that improvements in the acceleration capabilities of the paretic limb were associated with improvements in functional motor outcomes, and thus may offer evidence for motor control mechanisms underlying recovery in stroke rehabilitation. Consequently, successful interventions should employ exercise and feedback structures that focus on dynamic alterations of acceleration rates to enhance temporal control of paretic limb movements and improve clinical outcomes.

## Data Availability

The raw data supporting the conclusions of this article will be made available by the authors, without undue reservation.
